# Identification and Validation of Reference Genes for Expression Analysis in Nitrogen-Fixing Bacteria under Environmental Stress

**DOI:** 10.3390/life12091379

**Published:** 2022-09-05

**Authors:** Dylan Parks, Christian Peterson, Woo-Suk Chang

**Affiliations:** Department of Biology, University of Texas at Arlington, 501 S. Nedderman Dr., Arlington, TX 76019, USA

**Keywords:** housekeeping genes, qRT-PCR, gene expression, *Bradyrhizobium diazoefficiens*, nitrogen-fixing bacteria, environmental stress

## Abstract

Reference genes, also referred to as housekeeping genes (HKGs), play an important role in gene expression analysis by serving as an internal control. These HKGs are usually involved in basic cellular functions and their expression should remain at relatively constant levels. Quantitative reverse transcription-polymerase chain reaction (qRT-PCR) has been used to measure gene expression. Since the normalization of gene expression data depends on baseline expression of HKGs, it is important to identify and verify true HKGs for the qRT-PCR analysis. The goal of this study is to identify and confirm HKGs in *Bradyrhizobium diazoefficiens*, a nitrogen fixing bacterium which forms a symbiotic relationship with soybean. By revealing such HKGs, the normalization of gene expression would be more robust, reliable, and consistent. Here, we analyzed previous gene expression data for *B. diazoefficiens* under multiple environmental conditions. As a result, we identified seven constitutively expressed genes among 8453 genes across all conditions. Their fold-change values were within a range of −1.25-fold < x < 1.25-fold. We adopted GeNorm, NormFinder, and comparative ∆Ct methods to rank the seven candidate genes based on their expression stability. To validate these potential HKGs, we measured their expression in various experimental conditions, such as heat, pH, and heavy metal stress. The HKGs that were found in *B. diazoefficiens* were also applied in closely related species by identifying their homologs.

## 1. Introduction

Rhizobia are soil-dwelling, Gram-negative bacteria that form a symbiotic relationship with leguminous plants. This relationship results in the formation of root nodules in which they fix nitrogen and in turn receive photosynthetic products. These bacteria are of great importance in the agricultural industry because when properly inoculated in farmland soil, they can improve crop yields and decrease the reliance on expensive and harmful chemical nitrogen fertilizers [1]. By performing biological nitrogen fixation (BNF), they convert atmospheric nitrogen into ammonia, a form of nitrogen that plants can readily use, and thus serve as essential components to successful farmland soil. These rhizobia could also provide an efficient fertilization alternative to traditional chemical fertilizers which eventually run off into nearby water sources, resulting in ecological damage such as eutrophication events [2]. To better understand nitrogen-fixing bacteria and their symbiotic relationship, much effort has been put forth to reveal their physiological and molecular responses to typical environmental stresses, such as oxidative, pH, heat, desiccation, and osmotic stress [3,4,5,6]. In order to characterize the change in metabolic processes under these environmental conditions, the expression of the genes that are involved in physiological responses must be measured.

One of the most common and reliable sources of gene expression analysis is quantitative Reverse Transcription-Polymerase Chain Reaction (qRT-PCR). In the qRT-PCR analysis, gene expression levels must be normalized against the typical levels of expression that are found in the organism. By using a reference gene for quantifying baseline expression levels, a more accurate measurement can be provided for the true expression level of other genes. The reference genes, typically known as housekeeping genes (HKGs), are constitutively expressed during any environmental or cellular conditions [7,8]. Typical HKGs are usually involved in fundamental cell functions such as structural support, various metabolic processes, and the cell cycle. Therefore, they tend to be ubiquitous in nature [7].

HKGs have been routinely identified and used in other biological systems, both prokaryotic and eukaryotic cells, and are vital for not only understanding essential life maintenance, but also normalization in gene expression analysis. These genes have been identified by numerous methods such as mathematic modeling or a meta-analysis comparing the expression from high-density oligonucleotide arrays [9,10]. With the increase in databases harboring mass amounts of gene expression data, it has become easier to perform large-scale analysis to identify potential HKGs [11]. When testing new experimental conditions, the expression of these reference genes may fluctuate slightly depending on the investigation, which could lead to bias or skewed data [12]. Therefore, the prospective candidates should be validated in multiple different conditions or treatments and demonstrate some degree of expressional consistency to be considered a reliable internal control for downstream analysis [13].

Not only is qRT-PCR a powerful technique to assess gene expression, but it also a suitable method to validate candidate HKGs. This technique measures the amplification of complementary DNA (cDNA) from RNA transcripts via fluorescent probes and provides an accurate depiction of true gene expression due to its high sensitivity and dynamic range [14]. qRT-PCR is preferred over Northern blot hybridization, a conventional gene expression analysis, because of its sensitivity and range as well as precision and efficiency [15,16]. The constitutive nature of the reference genes is of importance since it is the standard for which all gene expression data are normalized against, and thus the improper selection could lead to inaccurate results [17]. In particular, some researchers have advised against the use of traditional HKGs that were developed from other studies, as their expression could differ greatly between biological samples [18]. Additionally, HKGs which are constitutively expressed in all conditions for a given species do not exist in other species and, therefore, should be identified and validated prior to each experiment.

Here, we provide data on the key potential HKGs for *Bradyrhizobium diazoefficiens* USDA110, a nitrogen-fixing rhizobium, identified from multiple microarray analyses, and validated under other environmental conditions using qRT-PCR. To compare these HKGs across closely related species, homologs were also identified and examined from two photosynthetic bradyrhizobial strains, *Bradyrhizobium* sp. ORS278 and *Bradyrhizobium* sp. BTAi1. Both ORS278 and BTAi1 are of great interest because of their implication in the nodulation factor (NF) independent symbiotic pathway, which demonstrates a novel symbiotic engagement that could be applied to a wide range of hosts due to their primitive nature compared to the NF-dependent nodulation [19,20].

## 2. Materials and Methods

### 2.1. Bacterial Strain Growth Conditions

The wild-type strains of *Bradyrhizobium diazoefficiens* USDA110, *Bradyrhizobium* sp. ORS278, and *Bradyrhizobium* sp. BTAi1 were cultured in arabinose-gluconate (AG) media at pH 6.8 which contained 125 mg of Na_2_HPO_4_, 250 mg of Na_2_SO_4_, 320 mg of NH_4_Cl, 180 mg of MgSO_4_·7H_2_O, 10 mg of CaCl_2_, 4 mg of FeCl_3_, 1.3 g of 4-(2-hydroxyethyl)-1-piperazineethanesulfonic acid (HEPES), 1.1 g of 2-(N-morpholino) ethanesulfonic acid (MES), 1 g of yeast extract, 1 g of L-arabinose, and 1 g of D-gluconic acid sodium sulfate per L [21]. All of the strains were incubated aerobically at 30 °C with vigorous shaking at 200 rpm for 3 days. An appropriate amount (30 µg·ml^−1^) of chloramphenicol antibiotic was added to the media for *B. diazoefficiens*.

### 2.2. Selection of Candidate Reference Genes

Candidate reference genes were selected by filtering microarray expression data from multiple different treatments in *B. diazoefficiens* to obtain an output of genes with log2-fold change values within a window of 0.8 < x < 1.25 which is equivalent to between −1.25-fold and 1.25-fold changes. The program R studio (http://www.rstudio.com, accessed on 27 August 2015) was used to download 13 *B. japonicum* (former name of *B. diazoefficiens*) microarray GEO files from NCBI which included the following conditions (Table S1): bacteroid state vs. free-living, chemoautotrophic vs. arabinose-supplemented chemoautotrophic growth, chemoautotrophic vs. heterotrophic growth, heterotrophic vs. arabinose-supplemented autotrophic growth, low nitrogen vs. high nitrogen, 20 µM coumestrol exposure, 1 mM indole-acetic-acid (IAA) treatment, H_2_O_2_ fulminant shock, H_2_O_2_ prolonged exposure, minimal vs. rich media, paraquat fulminant shock, paraquat prolonged exposure, and osmotic stress [22,23,24,25,26]. The expression values for all genes were also converted to log2-fold change values, and satisfactory genes within the target window were selected.

### 2.3. HKG Homolog Identification

Homologs from the candidate genes that were identified in *B. diazoefficiens* were found in both *Bradyrhizobium* sp. ORS278 and *Bradyrhizobium* sp. BTAi1 by performing nucleotide BLAST analysis through NCBI. We selected the best hit with the following parameters: e-value cutoff 1 × 10^−5^, query coverage > 50%, and percent identity > 35%.

### 2.4. Heat Shock Analysis

*B. diazoefficiens* cultures were grown in AG media and incubated aerobically at 30 °C with vigorous shaking at 200 rpm until they reached the exponential phase with OD_600_ of 0.8–1.0. There were three replicates of 30 mL cultures that were heat shocked by incubation in a water bath at 42 °C for 30 min, while the other three replicates were maintained 30 °C. Then, the cells were harvested for RNA extraction as described below.

### 2.5. Heavy Metal Exposure

For copper exposure, *B. diazoefficiens* cultures (200 mL) were grown as described above until splitting into two sets as follows: set #1-three replicates of 30 mL for the copper treatment, set #2-three replicates for the control treatment. A total of six cultures were then washed by first being centrifuged for 10 min at 8000× *g* and resuspended in AG media. The cultures were centrifuged again to obtain a cell pellet and resuspended in 30 mL of modified AG media with a final concentration of 1 mM of CuSO_4_·5H_2_O. For the control treatment, it followed the same washing step with only AG media. All of the cultures were incubated for 12 h at 30 °C with vigorous shaking at 200 rpm before the cells were harvested for RNA isolation. For zinc exposure, we followed the same experimental steps except for using 1 mM ZnSO_4_·7H_2_O for the zinc treatment.

### 2.6. pH (Acid) Stress Analysis

*B. diazoefficiens* cultures were grown as described above until splitting into two sets as in the following: set #1-three replicates of 30 mL for low pH (i.e., pH 4), and set #2-three replicates for the control treatment. A total of six cultures were then washed as described above. The cultures were centrifuged to obtain a cell pellet and resuspended in 30 mL of AG media with either a modified pH of 4.0. or neutral pH (i.e., pH 7.0). All six cultures were incubated for 30 min at 30 °C with vigorous shaking at 200 rpm until cell harvest for RNA extraction.

### 2.7. Photosynthetic Bradyrhizobia Temporal Analysis

For both *Bradyrhizobium* sp. ORS278 and *Bradyrhizobium* sp. BTAi1, the cultures were grown in AG media as described earlier, but under 16 h light and 8 h dark cycle. We used this day-night temporal cycle since both strains are known to have photosynthetic capability. The cultures were harvested and the RNA was isolated during the middle of each temporal (day and night) point for qRT-PCR analysis.

### 2.8. RNA Isolation

For heat stress, heavy metal exposure, and pH stress, RNA isolation was performed as previously described [24]. All of the cultures were condensed by centrifugation for 10 min at 4 °C and 8000× *g* in a fixed-angle rotor. The cell pellet was then collected by decanting the supernatant. Total RNA was extracted using a hot phenol method as described previously [27]. The RNeasy mini kit (Qiagen, Germantown, MD, USA) and RNase-free DNase (Qiagen, Germantown, MD, USA) was used to purify the isolated RNA according to manufacturer’s protocol. The RNA quantity was calculated using a NanoDrop device (Thermo Scientific, Bedford, MA, USA) and the RNA quality was confirmed via gel electrophoresis with 0.8% agarose gels.

### 2.9. qRT-PCR Gene Expression Analysis

The qRT-PCR was performed with modification according to methods that were previously described [22]. Briefly, cDNA was synthesized in 25-μL reaction solution containing 1.5 μL of M-MLV reverse transcriptase (200 U/μL; Promega Corp., Madison, WI, USA), 2 μL of random hexamers (250 ng/μL; Invitrogen, Carlsbad, CA, USA), 5 μL of 2.5 mM dNTPs, and 1 μg of total RNA. Of the reaction solution, 1 μL of cDNA was used as a template for qRT-PCR. A total of 10 μL qRT-PCR reactions were prepared using 5 μL of 2 × All-in-One^TM^ qPCR Mix and 0.1 μL ROX Reference Dye by GeneCopoeia (Rockville, MD, USA), 1 μL at 0.2 μM each of forward and reverse primers, and 1 μL of cDNA template. qRT-PCR was performed by using ABI PRISM 7300 instrument (Applied Biosystems, Foster City, CA, USA) and following the GeneCopoeia qPCR protocol. Negative control reactions which lacked reverse transcriptase were used to check for DNA contamination. Gene-specific primers for the selected candidate reference genes were designed using Integrated DNA Technologies (Coralville, IA, USA) company software. The primers that were used for qRT-PCR and corresponding amplicon size are listed in Table S2. The *parA* (bll0631) gene, encoding a chromosomal partitioning protein, was used to normalize the expression of the candidate HKGs in this study [5,26]. All of the experiments were performed with three biological replicates, with three technical replicates for each biological replicate. Fold induction values were calculated based on the Pfaffl method [28]. 

### 2.10. Gene Expression Stability Analyses

Candidate reference gene expression stability was obtained by importing raw expression values for all of the proposed internal control genes into GeNorm software [14] to calculate the M-value, which provides the average pairwise variation for a specific gene compared to other reference genes under the environmental conditions. In addition to GeNorm software, NormFinder [10] and comparative ∆Ct [29] methods were performed to provide additional stability data for ranking the proposed control genes. 

## 3. Results

### 3.1. Candidate Reference Genes Reflect Typical HKGs

We retrieved 13 microarray-based gene expression datasets from NCBI to compare the expression of 8453 genes under 13 different environmental conditions (Table S2). We excluded other expression data such as RNA-Seq to eliminate potential bias due to different technologies, although the latter technology provides higher sensitivity for measuring the number of RNA transcripts than the former technology. Nevertheless, only a few studies have been conducted using RNA-Seq for genome-wide transcriptional profiling of *B. diazoefficiens* under environmental stresses.

By comparing the expression values of 8543 genes from 13 different treatments, we identified 7 candidate reference genes, which constitutively expressed across all conditions (Table 1). They were selected by filtering 13 microarray expression data in *B. diazoefficiens* to obtain an output of genes with log2-fold change values within a window of log2 (0.8) < x < log2 (1.25) which is equivalent to between −1.25-fold and 1.25-fold changes. The candidate genes have similar functionality to previous genes that were categorized as HKGs (Table 1). Many of these genes encode for proteins that are involved in major cellular processes such as metabolism and molecule transport; however, two genes (bll3109 and blr3561) do not fit typical HKG conformity.

Since electron transport is integral to common cellular metabolic processes, it makes sense that a few of these candidate HKGs belong to the oxioreductase protein family. blr6296 encodes a probable GDP-mannose 6-dehydrogenase which is an enzyme that is most likely involved in the breakdown of different sugars, but has been characterized to encode for the biosynthesis of alginate [30]. Blr6358 is annotated as a putative oxidoreductase protein, while bll6396 encodes a putative 2-dehydropantoate 2-reductase which might be responsible for pantothenate biosynthesis, a precursor to Coenzyme-A [31].

The other candidate HKGs are involved in the transferase class of enzymes. bll6306 encodes a probable glycosyltransferase, which belongs to a superfamily of transfer enzymes that are usually responsible for the transfer of a glycosyl group from a sugar to a carbohydrate chain [32]. bll8166 encodes a probable phospholipid N-methyltransferase which is involved in the biosynthesis of phosphatidylcholine, a methylated derivative of phosphatidylethanolamine, where both are essential phospholipids in cell membranes [33].

### 3.2. bll8166 Is the Most Constitutively Expressed Gene under the Environmental Conditions

**Figure 1:**
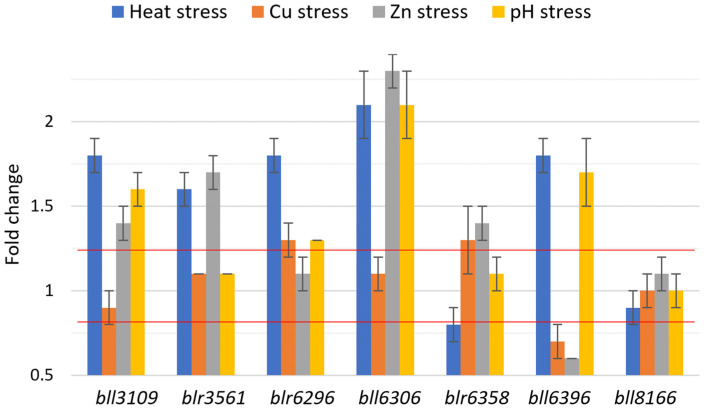
Fold changes of gene expression for the seven candidate genes during heat stress, heavy metal (Cu and Zn) exposure, and pH stress in *B. diazoefficiens* USDA110. Fold changes were calculated from gene expression values that were normalized to the HKG *parA*. The two red lines indicate the expression target window of 0.8 < x < 1.25 which has been used for selecting seven candidate genes from the microarray data. For the heat stress condition, the cultures were incubated at 42 °C for 30 min. For copper and zinc exposure, the cultures were resuspended in AG media containing either 1 mM CuSO_4_·5H_2_O or 1 mM ZnSO_4_·7H_2_O, respectively, and incubated at 30 °C for 12 h. For the pH (acid) stress condition, the cultures were resuspended in AG media with pH 4 and incubated at 30 °C for 30 min. Error bars represent standard errors of the mean with three biological replicates.

Among the seven candidate genes, bll8166 maintained its expression levels close to 1-fold within the target window of 0.8 < x < 1.25 under heat stress, copper and zinc exposure, and pH stress (Figure 1). We also adopted the GeNorm, NormFinder, and comparable ∆Ct methods to provide estimates of gene expression stability for the seven genes. bll8166 was found to have the lowest value, indicating that it is the most stable gene under the environmental stresses that were tested as well as the previously examined conditions. Interestingly, in the GeNorm analysis, all of the genes were below the cutoff value of 1.5 in the gene expression stability (Table 2). In general, an M-value of 1.5 or blow is considered to be reliable and stable gene expression [14].

### 3.3. bll8166 and blr6358 Serve as Reliable Reference Genes When Measuring Gene Expression under Heat, Heavy Metal, and pH Stresses

**Figure 2:**
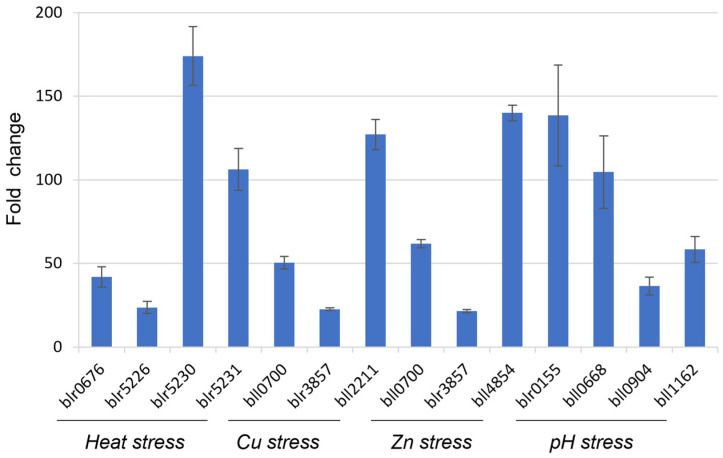
Fold changes of gene expression for stress-involved genes during heat stress, heavy metal exposure, and pH stress using bll8166 as a reference gene in *B. diazoefficiens* USDA110. Fold changes were calculated from the gene expression values that were normalized to the candidate gene bll8166. The function of the selected genes is as follows: blr0676, heat shock protein; blr5226, heat shock protein; blr5230, heat shock protein; blr5231, sigma32-like factor; bll0700, heavy-metal transporting P-type ATP transferase; blr3857, putative monooxygenase; bll2211, copper tolerance protein; bll4854, zinc metallopeptidase; blr0155, two-component response regulator; bll0668, glutathione synthetase; bll0904, two-component response regulator; bll1162, glutathione S-transferase. Error bars represent the standard errors of the mean with three biological replicates.

Both bll8166 and blr6358 genes were selected since they were the most constitutively expressed genes under the microarray conditions as well as heat, heavy metal, and pH stresses. Unlike bll8166, the stability values of blr6358 are median among the seven genes, which would provide a valuable counterpart for the comparison purpose in gene expression analysis. When measuring the expression of 12 stress-involved genes for either heat stress, heavy metal exposure, or pH stress via qRT-PCR, bll8166 proved to be a reliable reference gene for gene expression analysis since the 12 genes were highly upregulated when they were exposed to each relevant stress (Figure 2).

There were 12 genes that were selected based on their known cellular function in each stressful condition. For heat stress, three heat shock proteins and the corresponding sigma factor in the heat shock complex operon were chosen for their role in the stress response. For the heavy metal stress response, genes for copper tolerance and zinc metallopeptidase were chosen. Additionally, a gene encoding a putative monooxygenase is a homolog of a cobalt-zinc-cadmium resistance gene (czc) that is found in many soil bacteria including rhizobia, that is thought to mediate zinc resistance in heavy metal-polluted environments [34]. The heavy-metal transporting P-type ATP transferase gene was chosen for its role in efflux systems to transport metal ions across the cytoplasmic membrane in zinc-resistant bacteria [35]. For pH stress, two two-component response regulator genes, blr0155 and bll0904, were selected for gene expression analysis. These genes are homologs to those that are found in *Sinorhizobium meliloti* where they were shown to be a part of a two-component regulatory system that is involved in the acid tolerance response by pH sensing [36]. The other two genes that were chosen for the acid stress are involved in glutathione metabolism. Glutathione biosynthesis has been shown to be essential for bacterial response to acid tolerance in *Rhizobium tropici* species [37].

**Figure 3:**
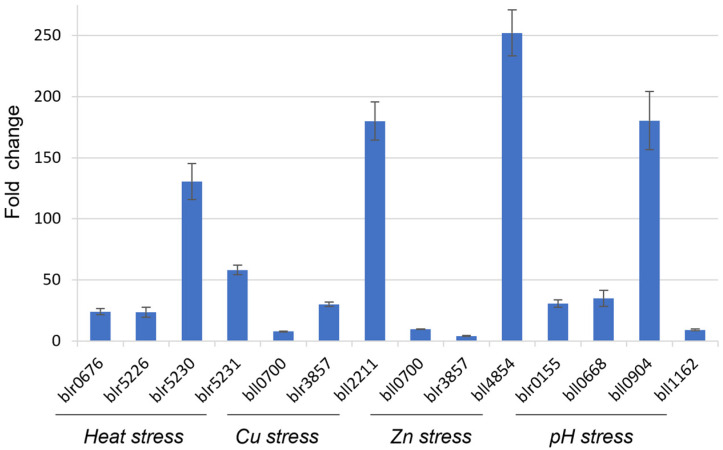
Fold changes of gene expression for stress-involved genes during heat stress, heavy metal exposure, and pH stress using blr6358 as a reference gene in *B. diazoefficiens* USDA110. Fold changes were calculated from gene expression values that were normalized to the candidate gene blr6358. The function of the selected genes is shown as described in the Figure 2 legend. Error bars represent the standard errors of the mean with three biological replicates.

The similar result was observed for another candidate gene blr6358 (Figure 3), although there were fluctuations in gene expression values compared to the bll8166-based qRT-PCR analysis (Table 3 and Table 4). This could be explained by independent sample preparation for each treatment. Overall, we observed the same trend of gene expression profiles among the selected genes in response to each environmental stress. This indicates that blr6358 can be also used as an internal control for qRT-PCR analysis under the environmental stress.

### 3.4. Six Homologs Found in ORS278 and BTAi1

Homologs for all the candidate genes in *B. diazoefficiens* were found in both *Bradyrhizobium* sp. ORS278 and BTAi1, except bll6396 which encodes a putative 2-dehydropantoate 2-reductase. For ORS278, three genes BRADO6535, encoding a hypothetical protein, BRADO0162, encoding a phospholipid N-methyltransferase, and BRADO6616, encoding a putative oxidoreductase, were constitutively expressed during the day and night cycle (Table 2). The target window of gene expression is a range of 0.8 < x < 1.25.

For BTAi1, the genes BBta_0193, encoding a phospholipid N-methyltransferase, BBta_2283, encoding a putative short-chain dehydrogenase/reductase, and BBta_1004, encoding a putative glycosyltransferase, were constitutively expressed under the temporal condition (Table 3). This result confirms that the homologs of bll8166 and blr6358 from *B. diazoefficiens* USDA110 are constitutively expressed in other closely related bradyrhizobia such as ORS278 and BTAi1, which further validates those two genes as suitable HKGs.

## 4. Discussion

Soil-dwelling nitrogen-fixing bacteria are an integral component of not only microbial ecosystems in native topsoil, but also agricultural communities. These organisms provide an avenue of opportunity to supply leguminous crops with a biological source of nitrogen instead of using chemical fertilizers [2]. By enhancing and optimizing biological nitrogen fixation (BNF) capabilities in the microbe-plant symbiosis, crop production could be vastly increased without diminishing ecological diversity in the surrounding environment or damaging the surrounding ecosystems [38].

To successfully apply and better understand this symbiosis, the exact mechanisms of the interaction must be defined on a molecular level. A reliable assessment of the expression of particular genes of interest could reveal insight into these microbial processes. Thus, it can be argued that normalization of gene expression data could be the most important aspect, especially when performing downstream analyses [39]. The selection of a suitable reference gene or a set of reference genes is the most reliable way to accurately gauge the expression of a particular gene of interest in a given experiment [14,37]. This technique has been used in a wide range of studies from microbes to humans, even in plants, which proves its versatility and reliability in multiple systems [40,41,42].

In this study, seven candidate reference genes were selected by comparing the expression values from a large bank of microarray-based expression dataset. Their fold change displayed constitutive expression levels under all conditions. Most of these genes fit the typical conformity of previously described internal control genes as they are necessary for housekeeping function such as cell growth and survival (e.g., metabolism, transport of molecules, and cell cycle). However, there were unexpected results, such as the gene bll3109, which is speculated as a calcium-binding protein, probably involved in signal transduction pathways, and blr3561, a hypothetical protein. Although blr3561 is characterized as a hypothetical protein, it contained a bacterial SH3 domain which has been predicted to be involved in bacterial cell wall binding and recognition, and possibly metal binding [43]. It would make sense for a calcium-binding protein or a metal-binding protein to be constantly expressed if it plays a role in maintaining homeostatic conditions within the cell.

Among the seven candidate genes, bll8166 showed the most consistent and stable expression characteristics, judging by fold change values under heat, metal, and pH stress conditions as well as the gene stability study using GeNorm, NormFinder, and comparative ∆Ct methods. The previous HKG *parA* (bll0631) that has been widely used in nitrogen fixing bacteria could be compared to bll8166 in order to rank for the gene expression stability. However, this gene did not pass our initial screening among the 13 microarray gene expression dataset with the target window of 0.8 < x < 1.25 for gene expression. This means that *parA* has been differentially expressed under one or more environmental conditions that are listed in Table S1. Thus, we suggest that bll8166 is superior than bll0631 as a reference gene for the gene expression study in rhizobia at least under abiotic stress.

To validate two HKGs bll8166 and blr6358, we selected 12 genes that are known to be expressed (Figure 2 and Figure 3) under the given conditions and measured their expression values using bll8166 or blr6358 as a reference gene. As expected, the expression of all of genes was induced more than 2-fold, ranging from 4.1- to 252.1-fold induction. Interestingly, we observed huge variabilities, although an overall trend is consistent. Particularly, when we compared the fold-change values of 12 genes between bll8166-based (Figure 2) and blr6358-based (Figure 3) results, several genes showed more than two times higher gene expression compared to one another. Included are bll0700, blr3857, blr0155, bll0668, bll0904, and bll1162. One potential explanation for this discrepancy could be the physiologically different cellular status when the cells were harvested for RNA isolation. We also postulate that different levels of induction could be due to other regulatory proteins (e.g., weak induction vs. strong induction). Additionally, it is noted that bll8166-based gene expression data might be more reliable than blr6358-based results because the former showed more stable and consistent gene expression patterns.

Although most of the genes proved to be constitutively expressed under the environmental stresses that were examined in the study, bll6306, encoding a probable glycosyl transferase, showed its fold change greater than two-fold under heat, zinc, and pH stresses (Figure 1). This result indicates that empirical proof could be necessary for any HKGs under a particular environmental condition. Among the seven genes, bll8166 and blr6358 maintained steady expression under heat, pH, and metal stresses. These conditions were chosen not only because of their absence from the original microarray dataset, but also their significance in terms of stressor types that *B. diazoefficiens* normally encounters in their micro-environment. Since *B. diazoefficiens* shares close homology with other nitrogen-fixing bacteria that inhabit the soil and form symbiotic relationships with leguminous plants, the candidate HKGs that were identified in the current study could possibly be applied to these bacterial relatives. Studies on *Sinorhizobium meliloti, Mesorhizobium loti,* or other members of the Bradyrhizobiaceae family could benefit from adopting homologs of the seven internal control genes for normalizing gene expression data.

Heat tolerance is a major factor in the survivability of microorganisms and they all proliferate within a specific temperature range. The optimum growth temperature for *B. diazoefficiens* is between 25 °C to 30 °C and will successfully nodulate and fix nitrogen in this range [44]. However, if the temperature rises above the optimum range, there are complications in the symbiotic process including root hair formation, differentiation into bacteroid, and nodule development, resulting in defect or delay in nodulation. If it surpasses 42 °C, the nodulation of soybean is significantly inhibited [3]. The gene *rpoE2*, encoding a putative extracytoplasmic function (ECF) sigma factor, had been identified in *S. meliloti* and proposed to play a regulatory role in the bacterial response to heat stress [45].

Metals such as nickel, zinc, copper, and cadmium have been shown to be toxic to rhizobia when they are exposed under long-term conditions [46]. Much effort has been put forth to diagnose the underlying mechanisms of microbial responses to toxic metals in the soil [47,48,49]. One study has identified a *Bradyrhizobium* sp. (vigna) strain RM8 that not only shows tolerance to toxic nickel and zinc, but also reduces the amount of the respective metals in plant organs [50]. Although it is difficult to compare and validate ecotoxicology studies between the field and laboratory, more information is needed to consider the implications of heavy metal buildup in the soil [51]. One such method could be a gene expression study on metal-binding proteins using qRT-PCR with a reliable HKG to provide biochemical pathway information for metal tolerance.

Soil pH is an important aspect of crop fertility as it limits the nitrogen fixation process and survival of rhizobia [52]. A proper range of pH must be maintained for the majority of rhizobia as both alkaline and acidic soils result in growth deficiencies and much effort has been made to identify certain strains which may be more tolerant to a wider range of pH [53,54,55]. One strain of *Rhizobium tropici* species displayed exceptional acid tolerance, which is linked to glutathione metabolism and thus is involved in the activation of potassium efflux pumps to cope with various environmental stresses [35]. Bacterial cells also respond to pH stress via the participation of a particular sigma factors. In *S. meliloti*, the sigma factor RpoH1 played a vital role during low pH stress by regulating genes that code for heat shock and chaperone proteins [56].

Overall, understanding the underlying mechanisms of the heat, heavy metal, and pH stress response and genes that are involved in the overall stress response in nitrogen-fixing bacteria is of great interest as this knowledge could lead to an enhancement in the symbiotic process, benefitting agricultural practices.

## 5. Conclusions

There were seven candidate HKGs that were identified and validated for the use of normalizing gene expression data in *B. diazoefficiens*. By analyzing mass amounts of microarray data, using qRT-PCR to monitor gene expression under the environmental stresses, and measuring gene stability with GeNorm, NormFinder, and comparative ∆Ct methods, the genes bll8166 and blr6358 have proven to be prime candidates for use as internal controls to normalize expression data when performing qRT-PCR analysis. Specifically, bll8166 was shown to have the most stable and consistent expression under the given conditions. Further analysis under other environmental conditions as well as biotic stress (e.g., plant hosts, viruses, and other antagonistic microorganisms) would provide extensive effectiveness of these genes to be used as internal controls for gene expression analysis.

## Figures and Tables

**Table 1:** Candidate reference genes that were obtained from microarray expression data.

Gene (Locus Name)	Function
bll3109	putative Ca-binding protein
blr3561	hypothetical protein
blr6296	probable GDP-mannose 6-dehydrogenase
bll6306	probable glycosyl transferase
blr6358	putative oxidoreductase protein
bll6396	putative 2-dehydropantoate 2-reductase
bll8166	probable phospholipid N-methyltransferase

**Table 2:** Gene expression stability rankings of the 7 candidate genes in *B. diazoefficiens* USDA110 under all conditions using different stability measurement methods.

Rank	GeNorm ^a^	NormFinder ^a^	Comparative ∆Ct ^b^
1	bll8166 (0.882)	bll8166 (0.298)	bll8166 (1.146)
2	bll6396 (0.907)	blr6296 (0.339)	blr6296 (1.210)
3	blr6296 (0.928)	bll6396 (0.370)	bl16396 (1.218)
4	blr6358 (1.014)	blr6358 (0.374)	blr6358 (1.320)
5	blr3561 (1.064)	blr3561 (0.418)	blr3561 (1.396)
6	bll6306 (1.183)	bll6306 (0.419)	bll6306 (1.419)
7	bll3109 (1.243)	bll3109 (0.622)	bll3109 (2.149)

^a^ Stability values are shown in parenthesis for each candidate gene. Lower values indicate higher stability. ^b^ Values represent the mean standard deviation of mean ∆Ct which are calculated from six paired comparisons for each gene (a total of six combinations for each gene). Lower values indicate higher stability.

**Table 3:** Fold changes of homologous genes in ORS278 for the HKGs between day and night.

Gene Homolog ^a^	Function	Fold Change ^b^
BRADO7074 (bll3109)	putative virulence factor MviN-like protein	0.3 ± 0.8
BRADO7042 (bll6306)	putative glycosyl transferase, group 1	0.6 ± 0.2
BRADO6535 (**blr3561**)	hypothetical protein	0.8 ± 0.3
BRADO0162 (**bll8166**)	phospholipid N-methyltransferase	1.0 ± 0.1
BRADO6616 (**blr6358**)	putative oxioreducatase; putative glucose/ribitol oxioreductase	1.1 ± 0.0
BRADO3450 (blr6296)	hypothetical protein	1.6 ± 0.1

^a^ Gene names in parenthesis represent *B. diazoefficiens* USDA110 gene locus. Bold letters indicate homologs within the target expression range of 0.8 < x < 1.25. ^b^ Day replicates served as the control condition, while night replicates served as the treatment condition.

**Table 4:** Fold changes of homologous genes in BTAil for the HKGs between day and night.

Gene Homolog ^a^	Function	Fold Change ^b^
BBta_4186 (bll3109)	hypothetical protein	0.6 ± 0.2
BBta_0193 (**bll8166**)	phospholipid N-methyltransferase	1.0 ± 0.1
BBta_2283 (**blr6358**)	putative short-chain dehydrogenase/reductase	1.0 ± 0.1
BBta_1004 (**bll6306**)	putative glycosyltransferase, group 1	1.2 ± 0.1
BBta_7236 (blr3561)	hypothetical protein	1.6 ± 0.2
BBta_7353 (blr6296)	UDP-glucose 6-dehydrogenase	1.6 ± 0.3

^a^ Gene names in parenthesis represent *B. diazoefficiens* USDA110 gene locus. Bold letters indicate homologs within the target expression range of 0.8 < x < 1.25. ^b^ Day replicates served as the control condition, while night replicates served as the treatment condition.

## Data Availability

Not applicable.

## Supplementary Materials

The following supporting information can be downloaded at: https://www.mdpi.com/article/10.3390/life12091379/s1, Table S1: Experimental conditions for gene expression (microarray) studies and their GEO accession numbers. Table S2: List of gene-specific primers that were used for qRT-PCR and corresponding amplicon size.
