# Supplementary material for: Identification and Validation of Reference Genes for Expression Analysis in Nitrogen-Fixing Bacteria under Environmental Stress
Source: Life (Basel). 2022 Sep 5;12(9):1379. doi: 10.3390/life12091379 (PMC9505014; doi:10.3390/life12091379)
Supplement: Supplementary file 1 [file life-12-01379-s001.zip › life-1836627-supplementary.pdf]

Table S1. Experimental conditions for gene expression (microarray) studies and their GEO accession numbers.

Treatments	GEO Accession No.
Minimal media vs. rich media	GSE8033
NaCl (50 mM)	GSE8036
Bacteroid state vs. free living	GSE8042
Heterotrophy vs. arabinose-supplemented chemoautotrophy	GSE10295
Chemoautotrophy vs. heterotrophy	GSE10296
Chemoautotrophy vs. arabinose-supplemented chemoautotrophy	GSE10298
Paraquat fulminant shock (5 mM for 10 min)	GSE26236
Paraquat prolonged exposure (0.1 mM)	GSE26252
Cuomestrol (20 µM for 24 h)	GSE26380
H_2O_2 prolonged exposure (0.3 mM)	GSE26960
H_2O_2 shock (10 mM for 10 min)	GSE26961
Indole-3-acetic acid (1 mM)	GSE36913
Low nitrogen vs. high nitrogen	GSE66091

Table S2. List of gene-specific primers that were used for qRT-PCR and corresponding amplicon size.

Gene (Locus Name)	Forward Primer (5′-3′)	Reverse Primer (5′-3′)	Amplicon Size (bp)
bll3109	CCGATGTCAAACAGGTCAA	GCAATCCTGTGACCGAAAT	144
blr3561	CGTTTAACCTCAGCAGCTAT	TGGTCATCATCCCAATTGTC	136
blr6296	CGACCGTCCATCAGATATTC	GTCTTTCGGTAGGCATGAG	101
bll6306	GTTCTGGGAGTCGTGTAATC	ACCAAGTCAGACCATACCT	102
blr6358	TTGAAATGGGCCGAGCAA	CAAGAAACCCGCCATCCA	235
bll6396	GCTCGTCAAATCCTACCATAC	CCGACTTCCTCCGATAGAA	126
bll8166	CTGGATTCGCGATCCTTTG	TATTCGACCAGCGTCAGAT	186
blr0676	GATGTTCGAAGTGCCTGAT	CAGTTCACTTCATTGCTGTTC	162
blr5226	CATCATCATTCCCGACACC	CTTCAGTACCCGACCATTTG	152
blr5230	CCTGTGGCGATCAACTATC	CAGACAGGCGTTCTACATT	110
blr5231	CCGCGGAATATGGAGTATC	CCTGCTTCATTTCTTTCGC	138
bll0700	GCGAGACCTTCCATTTCTG	TCTCCTTGGCGAGGTATTT	278
blr3857	CACCTACGGTCGATCTTTC	CGAAATCGCCTGATACCTC	108
bll2211	GATCAACAAGCTCGACCAAG	CGTCCCATAGTCCCATCAG	128
bll4854	CCAGTTCGTGATCAACCTC	CCGCCGCATAGAACATAAG	107
blr0155	GATCAAGACGCTGGAATGG	CCGCCTCCGAAATATTGAA	71
bll0668	CCCGCATCAATATCAAGGG	CTTGTCGGGCGTGTAATAG	92
bll0904	ATTCGCCGTGGTTGATTTG	GCGGGCTTGGAGAGATAAT	171
bll1162	CTACAAGCTGCTCGAATGG	GCTGGCTGTCGATGTATTT	146
bll0631	TCAACCTTCTGACGGTGAACGC	TGCAGCAATTGCGACAGACCTT	100
